# Impact of Recipient and Donor Obesity Match on the Outcomes of Liver Transplantation: All Matches Are Not Perfect

**DOI:** 10.1155/2016/9709430

**Published:** 2016-09-01

**Authors:** Eliza W. Beal, Dmitry Tumin, Lanla F. Conteh, A. James Hanje, Anthony J. Michaels, Don Hayes, Sylvester M. Black, Khalid Mumtaz

**Affiliations:** ^1^Department of General Surgery, Division of Transplantation, The Ohio State University Wexner Medical Center, Columbus, OH 43210, USA; ^2^Department of Anesthesiology and Pain Medicine, Nationwide Children's Hospital, Columbus, OH 43205, USA; ^3^Department of Internal Medicine, Division of Gastroenterology, Hepatology and Nutrition, The Ohio State University Wexner Medical Center, Columbus, OH 43210, USA; ^4^Department of Internal Medicine, Division of Pulmonary, Allergy, Critical Care and Sleep Medicine, The Ohio State University Wexner Medical Center, Columbus, OH 43210, USA

## Abstract

There is a paucity of literature examining recipient-donor obesity matching on liver transplantation outcomes. The United Network for Organ Sharing database was queried for first-time recipients of liver transplant whose age was ≥18 between January 2003 and September 2013. Outcomes including patient and graft survival at 30 days, 1 year, and 5 years and overall, liver retransplantation, and length of stay were compared between nonobese recipients receiving a graft from nonobese donors and obese recipient-obese donor, obese recipient-nonobese donor, and nonobese recipient-obese donor pairs. 51,556 LT recipients were identified, including 34,217 (66%) nonobese and 17,339 (34%) obese recipients. The proportions of patients receiving an allograft from an obese donor were 24% and 29%, respectively, among nonobese and obese recipients. Graft loss (HR: 1.27; 95% CI: 1.09–1.46; *p* = 0.002) and mortality (HR: 1.38; 95% CI: 1.16–1.65; *p* < 0.001) at 30 days were increased in the obese recipient-obese donor pair. However, 1-year graft (HR: 0.83; 95% CI: 0.74–0.93; *p* = 0.002) and patient (HR: 0.84; 95% CI: 0.74–0.95; *p* = 0.007) survival and overall patient (HR: 0.93; 95% CI: 0.86–1.00; *p* = 0.042) survival were favorable. There is evidence of recipient and donor obesity disadvantage early, but survival curves demonstrate improved long-term outcomes. It is important to consider obesity in the donor-recipient match.

## 1. Introduction

Currently, the two most common indications for liver transplant (LT) are alcohol and hepatitis C virus (HCV) related cirrhosis [1]. Nonalcoholic fatty liver disease (NAFLD) is a spectrum of diseases that includes bland steatosis and nonalcoholic steatohepatitis (NASH) and is currently the third most common indication for LT [1]. The frequency of NASH as an indication for LT has been increasing. In 2001, 1.2% of LTs were performed for NASH and by 2009 this figure had reached 9.6% [2]. Furthermore, experts predict that in the coming decades NAFLD will overtake alcohol and HCV as the most common indication for LT [1, 2]. Obesity is frequently associated with NAFLD. The prevalence of obesity in patients with NAFLD is reported to be between 30 and 100 percent [3] and the presence of NAFLD correlates with severity of obesity [4].

Research investigating the influence of recipient obesity on LT outcomes is controversial with conflicting results. Some studies report increased mortality [5–8] and decreased graft survival [7, 8] in obese recipients. In comparison, other studies have found no difference in overall survival between obese and nonobese recipients [9–12]. Moreover, it has been reported that survival of obese recipients is similar to nonobese recipients in living donor LT [13].

An important variable that has not been well studied in LT is the impact of donor obesity on posttransplant clinical outcomes. In pediatric LT recipients Perito et al. reported that children receiving adult donor livers with BMI > 35 had increased risk of graft loss and death. Pediatric recipients receiving a liver from an overweight or obese pediatric donor did not have an increase in graft loss or mortality [14]. Yoo et al. reported on the effect of donor obesity on recipient outcomes in adult LT. They determined that severe donor obesity or moderate steatosis did not influence short- or long-term outcomes including primary nonfunction of the graft, early retransplantation, and patient and graft survival [15]. However, no research has addressed the impact of donor-recipient obesity matching on clinical outcomes. We completed this study to determine the impact of recipient-donor obesity matching on LT outcomes after adjusting for various confounding variables. We hypothesize that matching of recipient-donor based on obesity has an association with overall patient survival after liver transplantation.

## 2. Methods

### 2.1. Data

The study was approved by the Institutional Review Board at Nationwide Children's Hospital with a waiver of individual consent (IRB14-00716). The United Network for Organ Sharing (UNOS) database was queried for first-time LT recipients who were ≥18 years of age transplanted between January 2003 and September 2013. Transplants from donation after cardiac death, split-livers, living donors, and combined transplants of liver with other organs were excluded. Transplants for acute liver failure were also excluded. We categorized the BMI of donors and recipients into nonobese (BMI < 30) and obese (BMI ≥ 30). Recipient BMI was collected at the time of transplantation. Outcomes of nonobese and obese recipients who received LTs from nonobese and obese donors were compared. Recipient-donor combinations included nonobese recipients (NOR) receiving grafts from nonobese donors (NOD), obese recipient-obese donor (OR-OD), obese recipient-nonobese donor (OR-NOD), and nonobese recipient-obese donor (NOR-OD). Nonobese recipients (NOR) receiving grafts from nonobese donors (NOD) were the reference group.

There were 51,556 patients eligible to be included in the study with data on both recipient and donor BMI. The primary outcome was overall patient survival. Secondary outcomes were 30-day, 1-year, and 5-year patient and graft survival and re-LT and length of hospital stay during index admission for LT. Arbitrarily short survival duration was assigned to patients or grafts surviving < 1 day. We also included donor macrovesicular steatosis, as a covariate previously described (≤15%, 20–30%, and >30%) in a supplemental model [16]. Based on this data a correlation between donor BMI and hepatic steatosis was studied.

Univariate survival analysis excluded 99 patients with unknown survival duration and multivariable analysis excluded 10,979 patients missing data on recipient, donor, or LT procedure characteristics. Multivariable subanalyses of conditional patient survival (1-year survival conditional on 30-day survival and 5-year survival conditional on 1-year survival) excluded 1,251 and 4,458 additional cases, respectively; multivariable subanalyses of conditional graft survival to 1 and 5 years excluded 1,922 and 5,682 additional cases, respectively; and multivariable analyses of length of hospital stay after LT and re-LT excluded 647 and 1,077 additional cases missing data on these respective outcomes.

### 2.2. Statistical Methods

Statistical analysis was performed using Stata/IC, version 13.0 (College Station, TX, StataCorp LP). Descriptive statistics were presented as means and standard deviations for continuous variables; and descriptive statistics for categorical variables were presented as counts and percentages. Cochran-Armitage tests for trends were used to describe the changing proportions of recipient-donor BMI combinations in LT performed over the study period. Comparisons among groups classified by recipient and donor obesity were performed using Chi-square tests for categorical variables and ANOVA for continuous variables. Kaplan-Meier curves and log-rank tests of survival functions were used to compare post-LT mortality and graft survival at 30 days, 1 year, and 5 years across four groups of patients defined by recipient and donor obesity. Recipient-donor obesity match was entered into a multivariable Cox proportional hazards model of overall patient survival adjusted for recipient, donor, and transplant characteristics to further examine differences in survival across recipient and donor obesity status. Due to a very high proportion of missing data, donor macrovesicular steatosis was added as a covariate in a supplemental model but was not included in the main analysis [16]. The multivariable Cox analysis was repeated for conditional patient survival outcomes and graft survival outcomes, whereas ordinary least-squares regression was used for the outcome of length of hospital stay (in days), and competing-risks regression was used for the outcome of re-LT, with mortality after LT considered a competing risk.

## 3. Results

### 3.1. Study Population

**Figure 1:**
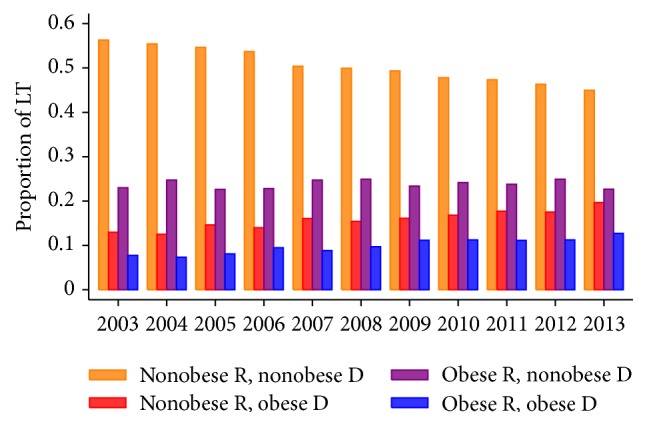
Proportions of liver transplants involving obese recipients and obese donors, by year of transplant (*N* = 51,556).

The characteristics of 51,556 LT recipients enrolled included in the study are summarized in Table 1. This analytic sample included 34,217 (66%) nonobese recipients (BMI < 30) and 17,339 (34%) obese recipients (BMI ≥ 30). The proportions of patients receiving an allograft from an obese donor (BMI ≥ 30) were 24% (8055/34217) and 29% (5044/17339), respectively, among nonobese and obese recipients. Cochran-Armitage tests for trends in proportions found significant increases in the proportions of LT involving OR and OD (*p* < 0.001) or NOR and OD (*p* < 0.001) and a significant decrease in the proportion of LT involving NOR and NOD (*p* < 0.001) over the study period. The trend in the proportion of LT involving OR and NOD was not statistically significant (*p* = 0.25) (Figure 1).

There were statistically significant differences in recipient age, gender, race, BMI, etiology of liver disease, diabetes mellitus (DM) status, portal vein thrombosis (PVT) at transplantation, and Model for End-Stage Liver Disease (MELD) score at the time of LT among the four categories of recipient-donor obesity matching. Similarly, donor age, gender, race, BMI, DM status, cold ischemia time (CIT), serum creatinine, and bilirubin were also significantly different among the four pairs. Obese recipients (OR) were more likely than nonobese recipients (NOR) to be white, to be male, to have a plausible diagnosis of NASH, and to have a history of DM. Obese donors (OD) were more likely to have a history of DM and hypertension and were older compared to NOD. Notably, the pair OR-NOD had the highest mean MELD score at LT (22.3 ± 10.2; *p* < 0.001) (Table 1).

Obese recipients (OR) had a higher mortality at 30 days (4%) compared to NOR (3%). However, 1-year (8% versus 9%), 5-year (10% versus 11%), and overall mortality (23% versus 25%) after LT was slightly less common among OR than in the modal group of NOR-NOD. Recipients with NOD tended to have longer length of hospital stay after LT (*p* = 0.04). Neither recipient nor donor obesity was associated with the likelihood of re-LT (*p* = 0.18) (Table 1).

### 3.2. Univariate Analysis

**Figure 2:**
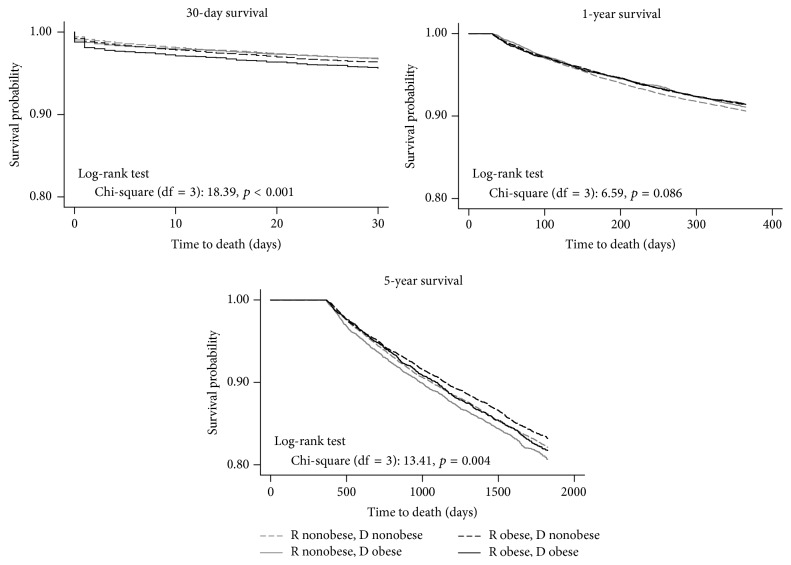
Kaplan-Meier 30-day, 1-year, and 5-year patient survival functions by recipient and donor obesity status (*N* = 51,556).

**Figure 3:**
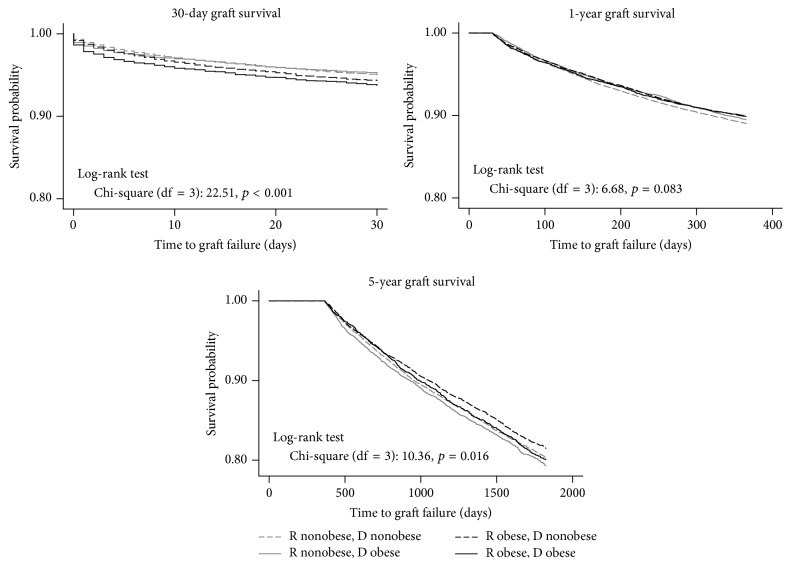
Kaplan-Meier 30-day, 1-year, and 5-year graft survival functions by recipient and donor obesity status (*N* = 51,556).

A log-rank test indicated no statistically significant differences in overall patient survival among the four groups stratified by the combination of recipient and donor obesity status (*p* = 0.05). Kaplan-Meier 30-day, 1-year, and 5-year patient survival curves stratified by the combination of recipient and donor obesity are shown in Figure 2. Mortality at 30 days was significantly increased in the OR-OD combination compared with the other three pairs (*p* < 0.001). The survival functions of OR and NOR cross over at the 3-month mark, with no statistically significant differences in survival between 30 days and 1 year after LT (log-rank test: *p* = 0.08). Long-term (5-year) survival, conditional on survival to 1 year, demonstrated an emerging survival advantage of the OR groups, particularly OR-NOD (*p* = 0.004). A similar pattern was observed in Kaplan-Meier 30-day, 1-year, and 5-year graft survival curves stratified by recipient and donor obesity, as shown in Figure 3. Early (30-day) graft failure was more common in the OR-OD group (*p* < 0.001), whereas 5-year graft survival favored the OR-NOD group (*p* = 0.01).

### 3.3. Multivariable Analysis

A multivariable Cox proportional hazards model of overall survival including recipient-donor obesity matching and potential confounding variables is presented in Table 2. After adjusting for covariates, the lowest mortality hazards were found among OR-NOD at 1 year (HR: 0.86; 95% CI: 0.78–0.94; *p* = 0.001) and 5 years (HR: 0.92; 95% CI: 0.85–1.00; *p* = 0.039) (Table 3(a)) and overall (HR = 0.91; 95% CI = 0.86–0.96; *p* < 0.001) relative to NOR-NOD (Table 2). Pairwise comparisons of OD and NOD within subgroups defined by recipient obesity revealed no independent contribution of donor obesity status to mortality hazard in the adjusted model. Donor steatosis data were available for 12,768 of the 40,478 patients included in Table 2. Among 4,928 obese donors with data on this variable, 225 (5%) had >30% steatosis, 808 (16%) had 20–30% steatosis, and 3,895 (79%) had <15% steatosis, as compared to 3% (224/7, 840) and 9% (673/7, 840) in the nonobese donor group (Chi-square: *p* < 0.001). Including donor steatosis in this model of overall survival indicated that there were no statistically significant differences by donor-recipient obesity match, but donor steatosis in the 20–30% range (HR = 1.16; 95% CI: 1.04, 1.29; *p* = 0.010) and in the >30% range (HR = 1.25; 95% CI: 1.03, 1.52; *p* = 0.026) was associated with increased mortality relative to <15% donor macrovesicular steatosis. Further modification of this model found no statistically significant interaction of steatosis with either donor age or cold ischemia time, suggesting that the latter factors did not modify the influence of donor liver steatosis on recipient survival (data not shown).

On multivariable regression analysis, mortality at 30 days was greatest for the OR-OD pair (HR: 1.38; 95% CI: 1.16–1.65; *p* < 0.001). Among the covariates described above, factors associated with increased risk of early mortality included PVT, older age, greater MELD score, and longer CIT. However, survival of the OR-OD group at 1 year (HR: 0.84; 95% CI: 0.74–0.95; *p* = 0.007) and overall survival (HR: 0.93; 95% CI: 0.86–1.00; *p* = 0.042) were favorable compared to NOR-NOD (Table 3(a)). Similarly, on multivariable regression analysis, graft loss at 30 days was greatest for the OR-OD pair (HR: 1.27; 95% CI: 1.09–1.46; *p* = 0.002), yet this difference was inverted by 1 year after LT, with OR-OD and OR-NOD having the lowest hazards of graft loss (HR: 0.83; 95% CI 0.74–0.93; *p* = 0.002 and HR: 0.86; 95% CI: 0.79–0.93; *p* < 0.001). At 5 years, conditional on 1 year of graft survival, no statistically significant differences remained in graft survival among groups defined by recipient and donor obesity (Table 3(b)).

Secondary outcomes of length of hospital stay and re-LT were analyzed using multivariable regression models (Table 3(c)). Predicted lengths of hospital stay were marginally shorter among OR-NOD and OR-OD pairs in comparison to the reference group of NOR-NOD. Adjusted hazards of re-LT were lowest in NOR-OD pair (HR = 0.81; 95% CI = 0.69–0.94; *p* < 0.006). Repeating the survival analysis with an obesity threshold of 40 kg/m^2^ did not show any significant differences from the analysis reported here with obesity threshold of 30 kg/m^2^ (data not shown). Considering underweight recipients, there were 732 underweight patients (BMI < 18) of whom 130 (18%) had an obese donor. Multivariable Cox analysis of 556 underweight respondents with complete covariate data (including all covariates shown in Table 2) found no significant association between donor obesity and mortality hazard in this subgroup (HR = 1.05; 95% CI = 0.71, 1.57; *p* = 0.802) (data not shown but available on request).

## 4. Discussion

In this study we demonstrated the impact of matching pairs of recipient-donor obesity on clinical outcomes of liver transplantation. We noted an important trend in LT with an increase in NOR-OD and OR-OD pairs and decrease in NOR-NOD. This is consistent with other literatures suggesting that an increasing proportion of LT donors and recipients are obese [15]. Interestingly, we found increased mortality in OR-OD pair in the first 30 days after LT. However, 1-year, 5-year, and overall survival in OR-OD were favorable compared to NOR-NOD. Outcomes information based on recipient and donor BMI may be helpful for physicians when accepting an offer for a specific patient. This emphasizes the importance of exploring outcomes based on body mass index or obesity status of both donors and recipients.

Research investigating the influence of recipient obesity on LT outcomes presents conflicting results with some demonstrating increased mortality and decreased graft survival in obese recipients and others demonstrating no difference in overall survival between obese and nonobese recipients [5–12]. The impact of donor obesity on LT outcomes has not been well studied. We found that mortality at 30 days is significantly increased in the OR-OD pair after adjusting for all confounding variables. However, this difference does not persist, and 1-year and overall survival favor the OR-OD pair. In contrast, the lowest mortality hazards were identified among OR-NOD at 1 year and 5 years and overall. Plausibly, graft loss is significantly increased in the OR-OD pair at 30 days, but this difference is inverted by 1 year after LT, with OR-OD and OR-NOD having the lowest hazards of graft loss. At 5 years, conditional on 1 year of graft survival, no statistically significant differences remained in graft survival among groups defined by recipient and donor obesity.

Our study found evidence of obesity disadvantage very early after LT. This survival disadvantage later reverts to reduced risk of mortality around 3 months after LT with obese recipients emerging as having improved 1-year and overall survival. Similarly, there is an obesity disadvantage in terms of early graft loss among OR-OD in the 30 days following LT. This difference in graft loss reverts in OR-OD and OR-NOD by 1 year. Due to study design we cannot directly assess the cause of early obesity disadvantage, but we speculate that early disadvantage in patients receiving livers from OD could be due to the increased fat content in OD grafts resulting in primary graft nonfunction. Other investigators have demonstrated that there is increased fat content in grafts obtained from obese donors [15, 17]. There is a correlation between severity of fatty infiltration in the donor liver and higher BMI. Marsman et al. reported lower 4-month graft survival in 59 patients who received a liver graft with up to 30% fat, compared with 57 who received livers without fatty infiltration, but found that this effect did not hold at the two-year mark, also demonstrating an early disadvantage [18]. A donor liver graft steatosis of <30 is recommended to be used for implantation. It is important to note that specific subpopulations may be at higher risk with donation from high BMI donors.

Our analysis demonstrates the lowest 1-year, 5-year, and overall mortality in the OR-NOD pair, relative to NOR-NOD. Several studies published this year reported equivalent survival among OR and NOR, without accounting for the obesity status of the donor. Using UNOS registry data from 2002 to 2013, Wong et al. reported that recipient obesity was not independently associated with worse outcomes for LT; however, presence of diabetes resulted in significantly lower survival [10]. In a meta-analysis, Saab et al. found that a combined analysis comparing 2275 OR to 72,212 NOR showed no difference in mortality. However, when they pooled four studies including 364 NOR and 128 OR with similar causes of liver disease they identified a reduction in survival in OR [12]. Using short-term outcomes of OR with University Health System Consortium (UHC) and Scientific Registry of Transplant Recipients (SRTR) data, Singhal et al. reported equivalent short-term outcomes including patient and graft survival between OR and NOR. However, donor obesity status was not accounted for in their analysis [11]. With improved graft and patient survival in OR-NOD, our findings are in direct contrast to these studies.

Obesity has been shown to be protective in patients in various clinical settings, including patients admitted to the intensive care unit (ICU) [19, 20], patients with severe sepsis [21, 22], and patients undergoing percutaneous coronary intervention [23]. Obese LT recipients are often critically ill and spend several days in the ICU. There are multiple hypotheses for the improved outcomes demonstrated in obese patients in these settings. It has been demonstrated that obesity leads to loss of tissue homeostasis and development of an inflammatory response [24, 25]. However, critical illness leads to the accumulation of alternatively activated M2 macrophages with a more anti-inflammatory role [25]. It has also been observed that critically ill obese patients with ARDS have reduced levels of inflammatory cytokines [26]. The shift to an anti-inflammatory milieu may partially explain the obesity advantage in LT patients. Another possible explanation relates to the nutritional reserves possessed by obese patients, which may help them tolerate the increased metabolic demands of critical illness [19].

Our study found evidence of shorter length of stay in the OR-OD pair and OR-NOD pair. Predicted lengths of hospital stay were marginally shorter among OR-NOD or OD compared to the reference group of NOR-NOD. Singhal et al. report that patients with BMI > 40 have increased post-LT length of stay in comparison to patients with BMI < 40 (9 versus 11 days, *p* < 0.0001) [11]. In a single-center study, Tanaka et al. report increased duration of overall hospital stay for patients with BMI > 40 when using conventional BMI calculations. Using a modified BMI calculation to account for fluid accumulation they report equivalent length of hospital stay for all groups [27]. In a single-center study Conzen et al. report equivalent length of stay between OR and NOR [7]. These are in contrast to our study, in which we found a decreased length of stay in OR who received donations from both NOD and OD.

Our analysis found a reduced adjusted hazard of re-LT in the NOR-OD pair as compared to the reference pair. This was consistent with Yoo et al. who investigated LT outcomes related to donor obesity and donor liver steatosis and identified no differences in early re-LT outcomes associated with donor obesity and donor liver steatosis. However, their analysis did not include donor obesity as a covariate. Yoo et al. suggest, and we agree, that larger donor liver size may compensate in some respects for increased fatty infiltration in donor livers from OD [15], which is supported by the findings of the current study.

Our analysis has several limitations related to the nature of collection and reporting of data in the UNOS database, which could include data entry errors, missing data, and omission of important data. A substantial number of patients were excluded because of missing recipient/donor BMI and other variables. Due to the impact of ascites on weight, the reliability of recipient BMI measurement in liver transplant recipients may not be very accurate [28]. Reporting of donor liver biopsy and steatosis, which was previously part of UNOS database, is no longer available. Despite these limitations, the study does draw from a large, multi-institutional registry database of transplant recipients and is generalizable. The large number of patients included in UNOS database allows for models adjusting for multiple confounding variables. Additionally, the large, multi-institutional nature of this study reduces potential bias observed in single-institution observational studies.

In conclusion, there is increasing trend of LT among NOR-OD and OR-OD pairs and decrease in NOR-NOD. We identified increased 30-day mortality and graft loss among OR-OD pairs; however, this relationship improved rather quickly and resulted in reduced mortality hazard at subsequent follow-up intervals of 1 year and 5 years and reduced graft loss at 1 year and equivalent graft loss at 5 years. Information on BMI based on outcomes may be helpful in selecting a donor for a specific recipient at the time of an organ offer. Holding consistent with obesity being protective, the match pair with the least hazard mortality was OR-NOD. Although we cannot determine causality of our findings due to study design, we have found intriguing donor-recipient interactions that may assist clinicians in important decisions with the need for further research to investigate the protective pathways associated with obesity in LT.

## Figures and Tables

**Table 1:** Descriptive statistics by recipient donor obesity (*n* = 51,556).

Covariates	Nonobese R		Obese^*∗*^ R		*p* ^*∗∗*^
	Nonobese D (*n* = 26162)	Obese^*∗*^ D (*n* = 8055)	Nonobese D (*n* = 12295)	Obese D (*n* = 5044)	
	*N* (%) or mean (SD)	*N* (%) or mean (SD)	*N* (%) or mean (SD)	*N* (%) or mean (SD)	
Male recipient	17707 (68%)	6177 (77%)	7967 (65%)	3661 (73%)	<0.001

Recipient age	53.4 (10.6)	54.0 (10.0)	54.3 (8.7)	54.7 (8.3)	<0.001

Recipient race					<0.001
White	18395 (70%)	5887 (73%)	9090 (74%)	3928 (78%)	
Black	2549 (10%)	794 (10%)	1096 (9%)	408 (8%)	
Other	5218 (20%)	1374 (17%)	2109 (17%)	708 (14%)	

Etiology					<0.001
Viral	7439 (28%)	2183 (27%)	3443 (28%)	1297 (26%)	
Cryptogenic	1446 (6%)	455 (6%)	946 (8%)	398 (8%)	
Autoimmune	3276 (13%)	881 (11%)	864 (7%)	305 (6%)	
NASH	811 (3%)	257 (3%)	1258 (10%)	587 (12%)	
Alcoholic	4442 (17%)	1575 (20%)	2093 (17%)	904 (18%)	
HCC	4897 (19%)	1681 (21%)	2338 (19%)	1072 (21%)	
Other	3850 (15%)	1021 (13%)	1351 (11%)	481 (10%)	

Recipient diabetes	5565 (22%)	1772 (22%)	3671 (30%)	1526 (31%)	<0.001

Acute rejection before discharge	1468 (6%)	484 (7%)	670 (6%)	286 (6%)	0.588

PVT at transplantation	1817 (7%)	533 (7%)	1011 (8%)	396 (8%)	<0.001

MELD score at LT	21.4 (10.1)	21.2 (10.0)	22.3 (10.2)	21.5 (9.9)	<0.001

Recipient BMI	24.8 (3.2)	25.3 (3.0)	34.5 (3.8)	34.7 (3.8)	<0.001

Male donor	15721 (60%)	4006 (50%)	7929 (64%)	2766 (55%)	<0.001

Donor age	40.4 (17.7)	45.5 (14.7)	41.2 (17.3)	45.7 (14.8)	<0.001
Donor race					<0.001
White	17356 (66%)	5160 (64%)	8409 (68%)	3304 (66%)	
Black	4159 (16%)	1634 (20%)	1964 (16%)	1052 (21%)	
Other	4647 (18%)	1261 (16%)	1922 (16%)	688 (14%)	

Donor diabetes	1976 (8%)	1440 (18%)	1002 (8%)	990 (20%)	<0.001

Donor hypertension	7507 (29%)	4013 (50%)	3723 (30%)	2591 (52%)	<0.001

Donor CMV positive	17273 (66%)	5416 (67%)	8043 (66%)	3314 (66%)	0.062

Donor HBV positive	1492 (6%)	444 (6%)	621 (5%)	250 (5%)	0.023

Donor HCV positive	920 (4%)	210 (3%)	473 (4%)	107 (2%)	<0.001

Cold ischemia time	7.0 (3.4)	7.1 (3.4)	7.2 (3.5)	7.2 (3.5)	<0.001

Donor creatinine	1.4 (1.5)	1.7 (1.8)	1.5 (1.5)	1.8 (1.8)	<0.001

Donor BMI	24.2 (3.3)	34.9 (4.9)	24.6 (3.2)	35.4 (5.3)	<0.001

Death after LT	6470 (25%)	1913 (24%)	2836 (23%)	1166 (23%)	0.001
Death within 1–30 days	848 (3%)	253 (3%)	440 (4%)	218 (4%)	<0.001
Death past 30 days, within 1 year	2175 (9%)	622 (8%)	931 (8%)	372 (8%)	0.029
Death past 1 year, within 5 years	2631 (11%)	815 (11%)	1114 (10%)	461 (10%)	0.004

Retransplant	1180 (5%)	326 (4%)	571 (5%)	214 (4%)	0.179

Length of hospital stay post-LT	16.7 (22.5)	16.1 (21.8)	16.8 (24.2)	16.1 (20.4)	0.046

Survival time	1275 (1049)	1157 (991)	1235 (1034)	1137 (995)	<0.001

^*∗*^Obesity defined as BMI ≥ 30 kg/m^2^.  ^*∗∗*^Chi-square test for categorical variables and ANOVA for continuous variables.

**Table 2:** Multivariable Cox proportional hazards regression of survival after liver transplantation (*n* = 40,478).

Covariates	HR	95% CI	*p*
Male recipient	0.94	(0.90, 0.99)	0.010

Recipient age	1.02	(1.01, 1.02)	<0.001

Recipient race			
White	Ref.		
Black	1.28	(1.20, 1.37)	<0.001
Other	0.84	(0.79, 0.89)	<0.001

Etiology			
Viral	Ref.		
Cryptogenic	0.73	(0.67, 0.80)	<0.001
Autoimmune	0.67	(0.61, 0.74)	<0.001
NASH	0.72	(0.65, 0.80)	<0.001
Alcoholic	0.82	(0.77, 0.87)	<0.001
HCC	1.13	(1.06, 1.20)	<0.001
Other	1.12	(1.05, 1.20)	0.001

Recipient diabetes	1.21	(1.16, 1.27)	<0.001

Acute rejection before discharge	1.14	(1.05, 1.24)	0.001

PVT at transplantation	1.23	(1.15, 1.33)	<0.001

MELD score at LT	1.02	(1.02, 1.03)	<0.001

Male donor	1.01	(0.96, 1.05)	0.790

Donor age	1.01	(1.01, 1.01)	<0.001

Donor race			
White	Ref.		
Black	0.99	(0.94, 1.06)	0.845
Other	1.17	(1.11, 1.24)	<0.001

Donor diabetes	1.09	(1.02, 1.17)	0.010

Donor hypertension	0.99	(0.94, 1.05)	0.845

Donor CMV positive	1.03	(0.98, 1.08)	0.224

Donor HBV positive	1.06	(0.97, 1.16)	0.191

Donor HCV positive	1.16	(1.04, 1.29)	0.008

Cold ischemia time	1.01	(1.01, 1.02)	<0.001

*BMI of recipient (R) and donor (D)* ^*∗*^			
Nonobese R, nonobese D	Ref.		
Nonobese R, obese D	0.98	(0.92, 1.04)	0.547
Obese R, nonobese D	0.91	(0.86, 0.96)	<0.001
Obese R, obese D	0.93	(0.86, 1.00)	0.042

^*∗*^Obesity defined as BMI ≥ 30 kg/m^2^.

**(a):** 

	30-day survival^a^ (*n* = 40,478)			1-year survival^a^ (if they survived to 30 days; *n* = 39,227)			5-year survival^a^ (if they survived to 1 year; *n* = 36,020)		
	HR	95% CI	*p*	HR	95% CI	*p*	HR	95% CI	*p*
R nonobese, D nonobese	Ref.			Ref.			Ref.		
R nonobese, D obese	1.02	(0.86, 1.21)	0.823	0.91	(0.82, 1.01)	0.076	1.03	(0.94, 1.13)	0.493
R obese, D nonobese	1.09	(0.94, 1.25)	0.247	0.86	(0.78, 0.94)	0.001	0.92^b^	(0.85, 1.00)	0.039
R obese, D obese	1.38^b,c^	(1.16, 1.65)	<0.001	0.84	(0.74, 0.95)	0.007	0.90^b^	(0.81, 1.02)	0.088

^a^Cox proportional hazards model. ^b^Statistically significant difference relative to “R nonobese, D obese” group. ^c^Statistically significant difference relative to “R obese, D nonobese” group.

All models are adjusted for D and R gender, D and R race, D and R age, R diagnosis, D and R diabetes history, D and R serum creatinine, D and R bilirubin, R INR, albumin, and MELD score at LT, and D SGOT and SGPT, acute rejection, PVT, and cold ischemia time. LT = liver transplantation, R = recipient, D = donor, HR = hazard ratio, and CI = confidence interval.

**(b):** 

	30-day survival^a^ (*n* = 40,478)			1-year survival^a^ (*n* = 38,556)			5-year survival^a^ (*n* = 34,796)		
	HR	95% CI	*p*	HR	95% CI	*p*	HR	95% CI	*p*
R nonobese, D nonobese	Ref.			Ref.			Ref.		
R nonobese, D obese	0.97	(0.85, 1.11)	0.703	0.89	(0.81, 0.98)	0.019	1.00	(0.92, 1.09)	0.980
R obese, D nonobese	1.14^b^	(1.02, 1.28)	0.018	0.86	(0.79, 0.93)	<0.001	0.93	(0.86, 1.01)	0.077
R obese, D obese	1.27^b^	(1.09, 1.46)	0.002	0.83	(0.74, 0.93)	0.002	0.90	(0.81, 1.01)	0.071

^a^Cox proportional hazards model. ^b^Statistically significant difference relative to “R nonobese, D obese” group. All models are adjusted for D and R gender, D and R race, D and R age, R diagnosis, D and R diabetes history, D and R serum creatinine, D and R bilirubin, R INR, albumin, and MELD score at LT, and D SGOT and SGPT, acute rejection, PVT, and cold ischemia time.

LT = liver transplantation, R = recipient, D = donor, HR = hazard ratio, and CI = confidence interval.

**(c):** 

	Overall patient survival^a^ (*n* = 40,478)			Days of hospital stay post-LT^b^ (*n* = 39,831)			Retransplantation^c^ (*n* = 39,401)		
	HR	95% CI	*p*	*b*	95% CI	*p*	SHR	95% CI	*p*
R nonobese, D nonobese	Ref.			Ref.			Ref.		
R nonobese, D obese	0.98	(0.92, 1.04)	0.547	−0.58	(−1.22, 0.05)	0.071	0.81	(0.69, 0.94)	0.006
R obese, D nonobese	0.91^d^	(0.86, 0.96)	<0.001	−0.64	(−1.18, −0.10)	0.021	1.11^d^	(0.98, 1.26)	0.096
R obese, D obese	0.93	(0.86, 1.00)	0.042	−0.93	(−1.68, −0.17)	0.016	0.91^e^	(0.76, 1.09)	0.314

^a^Cox proportional hazards model. ^b^Ordinary least-squares regression model. ^c^Competing-risks regression model with mortality as a competing risk. ^d^Statistically significant difference relative to “R nonobese, D obese” group. ^e^Statistically significant difference relative to “R obese, D nonobese” group. All models are adjusted for D and R gender, D and R race, D and R age, R diagnosis, D and R diabetes history, D and R serum creatinine, D and R bilirubin, R INR, albumin, and MELD score at LT, and D SGOT and SGPT, acute rejection, PVT, and cold ischemia time. LT = liver transplantation, R = recipient, D = donor, HR = hazard ratio, *b* = unstandardized coefficient, SHR = subhazard ratio, and CI = confidence interval.

## Abbreviations

BMI: Body mass index
HCV: Hepatitis C virus
LT: Liver transplant
NOR-NOD: Nonobese recipient-nonobese donor
NOR-OD: Nonobese recipient-obese donor
NOR: Nonobese recipient
NOD: Nonobese donor
NAFLD: Nonalcoholic fatty liver disease
NASH: Nonalcoholic steatohepatitis
OR-NOD: Obese recipient-nonobese donor
OR-OD: Obese recipient-obese donor
OR: Obese recipient
OD: Obese donor
re-LT: Re-liver transplant
UNOS: United Network for Organ Sharing.
